# Three-dimensional reconstruction of surface nanoarchitecture from two-dimensional datasets

**DOI:** 10.1186/2191-0855-4-3

**Published:** 2014-01-10

**Authors:** Veselin Boshkovikj, Hayden K Webb, Vy T H Pham, Christopher J Fluke, Russell J Crawford, Elena P Ivanova

**Affiliations:** 1 Faculty of Life and Social Sciences, Swinburne University of Technology, PO Box 218, Hawthorn, Victoria 3122, Australia; 2 Centre for Astrophysics and Supercomputing, Swinburne University of Technology, PO Box 218, Hawthorn, Victoria 3122, Australia

**Keywords:** Three-dimensional visualization, Scanning electron microscopy, Atomic force microscopy, Surface topographical analysis

## Abstract

The design of biomaterial surfaces relies heavily on the ability to accurately measure and visualize the three-dimensional surface nanoarchitecture of substrata. Here, we present a technique for producing three-dimensional surface models using displacement maps that are based on the data obtained from two-dimensional analyses. This technique is particularly useful when applied to scanning electron micrographs that have been calibrated using atomic force microscopy (AFM) roughness data. The evaluation of four different surface types, including thin titanium films, silicon wafers, polystyrene cell culture dishes and dragonfly wings confirmed that this technique is particularly effective for the visualization of conductive surfaces such as metallic titanium. The technique is particularly useful for visualizing surfaces that cannot be easily analyzed using AFM. The speed and ease with which electron micrographs can be recorded, combined with a relatively simple process for generating displacement maps, make this technique useful for the assessment of the surface topography of biomaterials.

## Introduction

Advances in microscopic technology have revolutionized the way objects can be perceived on the nanoscale. Powerful instruments such as the scanning electron microscope (SEM) provide the ability to rapidly characterize samples using high magnification, producing two-dimensional (2D) image representation of the samples (Cizmar et al., 2008; Schatten, 2011), making it highly a suitable analytical technique in both biological and materials sciences (Coelho et al., 2009; Kang et al., 2009). The main drawback of the technique however, is that the data obtained is limited to two dimensions (i.e. height and width, but not depth) which can often limit the extent of understanding that can be obtained regarding the morphology and topographical features of many samples.

In order to perform an in-depth characterization of material surface structures and architecture, a number of different methods have emerged for the purposes of 3D surface visualization. Atomic force microscopy (AFM) is perhaps the most useful of these techniques, as it can be employed to generate topographical maps of a surface with sensitive and accurate measurement in the depth dimension (Binnig et al., 1986). This data can be employed to reconstruct three-dimensional surface models (Schift et al., 2009). AFM surface scans are physical in nature, in that the AFM tip physically interacts with a sample surface, producing 3D coordinates. Some drawbacks of the technique are that in cases where the surface topography is extremely complex or heterogenous in its mechanical properties, the resulting data may not be accurate. In addition, AFM scans are typically only able to sample a small section of the surface, and take a relatively long time to obtain a representation of a surface. In contrast, SEM can be used to sample much larger fields of view with high resolution over a shorter period of time. The main drawback of SEM is that unlike AFM, the height values are not measured in the depth dimension, and so it cannot be readily utilized for the 3D visualization of samples. To overcome this limitation, stereo scanning electron microscopes have been developed (Marschall et al., 2000). In this technique, two electron beams on two different angles are focused at the same point on a sample and the three-dimensional coordinates are measured, based on the data obtained from the two different perspectives (Ostadi et al., 2011). The ability to accurately compute the 3D points strongly depends on the ability to accurately match the two images (Samak et al., 2007).

**Figure 1:**
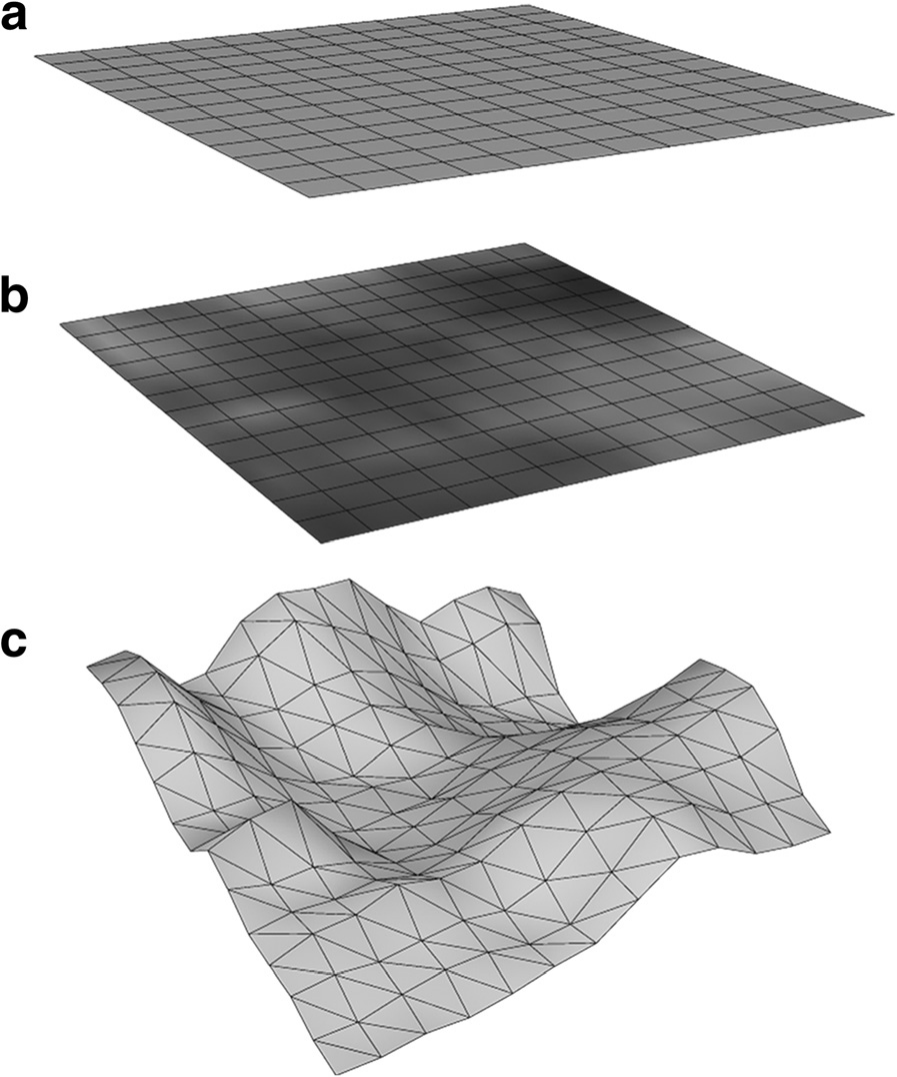
**Application of displacement maps.** Each polygonal object (e.g. a plane) consists of three-dimensional points (vertices) that are connected with lines (edges) **(a)**. Each pixel is assigned a relative height value based on their grey-scale values; black is assigned the lowest value of 0 and white the highest value of 1. **(b)**. During the displacement map geometry conversion each vertex is translated in 3D space according to its assigned height value, thus transforming the original, planar 2D image into a new 3D geometry **(c)**.

Displacement maps are commonly used for the application of high-resolution information using low-resolution models (Szirmay-Kalos and Umenhoffer, 2008; Lu et al., 2009; Jang and Han, 2012). These maps are based on gray scale (alpha) information, and as such they allow the transformation or deformation of 3D objects according to information collected from 2D images, where black represents the lowest point in space and white represents the highest point (Figure 1). Displacement maps assign each pixel in an image a height value based on its alpha value, determined according to an arbitrary height range. For example, the pixels in an image may be assigned height values ranging between 0 (black) and 1 (white). In this case, if the alpha value of a given point is greater than 0, the point is then translated perpendicular to the 2D image plane. When each point has been translated relative to each other in this manner, a *proportionally* accurate 3D surface structure is produced (Figure 1c). In most cases however, displacement maps are only produced for aesthetic purposes; no absolute height values can be generated (Dmitriev and Makarov, 2011). Given that most SEM systems produce greyscale images by default, electron micrographs are a prime candidate for the production of displacement maps, and when coupled with an appropriate height calibration procedure, 3D models can be produced for a surface that contain relatively accurate height values.

The aim of this study was to develop and evaluate a technique for the effective three-dimensional reconstruction of the nanoarchitecture of a surface, based on two-dimensional electron micrographs. This technique combines the rapid data collection capability of SEM with the accurate three-dimensional measurement ability of AFM. As a result, large areas of material surfaces can be rapidly analyzed and subsequently presented as high-resolution 3D models.

## Materials and methods

### Data collection

#### Sample preparation

Four substrata surfaces were analyzed using SEM to demonstrate the versatility of the technique: 150 nm-thick thin titanium films (prepared as described previously (Wang et al., 2008), dragonfly (*Hemianax papuensis*) wings, standard polystyrene petri plates (Cellstar, Greiner Bio-One) and silicon wafers. Prior to analysis, all samples were rinsed with 70% ethanol and then MilliQ H_2_O, except in the case of the dragonfly wings, which were rinsed with water, as ethanol would damage the waxy surface structures of the wing. The dragonfly wings and small excised pieces of polystyrene were also coated with ~10 nm of gold using a Dynavac CS300 prior to electron microscopy in order to make the surfaces conductive (Mitik-Dineva et al., 2009; Truong et al., 2009).

#### Scanning electron microscopy (SEM)

The electron micrographs of all four sample types were recorded using a FESEM (ZEISS SUPRA 40VP) at 3 kV at 70000× magnification. In addition, titanium films were analysed at 30000×, 90000× and 150000× magnifications. All samples were initially viewed at lower magnification in order to identify suitable regions of the surface for analysis, prior to analysis at higher magnification.

#### AFM surface calibration

All AFM scans were conducted using an Innova scanning probe microscope (Veeco, U.S.A.). Scans were performed in tapping mode at ambient temperature and pressure, using silicon cantilevers (MPP-31120-10, Veeco, U.S.A.) with a spring constant of 0.9 N m^-1^ and a resonance frequency of approximately 20 kHz. Scanning was performed perpendicular to the axis of the cantilever at a scan speed of 1 Hz. Initially, 10 μm × 10 μm fields of view were scanned in order to identify suitable analysis regions of the surface, prior to analysis at higher resolution. Scan areas were selected to closely match with the resolution of electron micrographs, as the distance between individual sampling points can affect the calculated roughness parameters (Brune et al., 1997; Crawford et al., 2012).

### Three-dimensional visualization of SEM images

The 3D visualization of SEM images was realized in the 3D animation software package Autodesk Maya® (http://usa.autodesk.com/maya/). Maya’s 3D modeling and shading/texturing capabilities were used for construction of displacement map from the SEM images and conversion into 3D polygonal geometry.

#### Construction of displacement maps

Displacement maps were constructed according to the following procedure: A polygonal plane was created as a reference point for the creation of 3D images from the electron micrographs. By default, the plane was a two-dimensional object. The resolution and dimension attributes of each plane were set according to the corresponding SEM image being used for modeling. The resolution of each plane was adjusted to match that of the SEM image, and the x- and y-dimensions (i.e. height and width) were set to correspond with those of the actual areas being analyzed. Data representing the depth dimension was extracted from the alpha values of each pixel in the SEM images. A simple script was developed using Python programming language (Python Software Foundation) in order to obtain the height values at each point of the surface topography. The script recorded the alpha values of each pixel as a relative translation attribute value for each vertex. The data was then stored in an empty comma-separated values file (.csv).

Roughness data calculated from AFM scans were used to calibrate the depth dimension scale of the displacement maps. The average value of the .csv files were set to match the average roughness (*R*_a_) determined for each of the corresponding sample surfaces, and all other values were scaled proportionate to this value.

#### Conversion of displacement maps into 3D polygonal geometry

**Figure 2:**
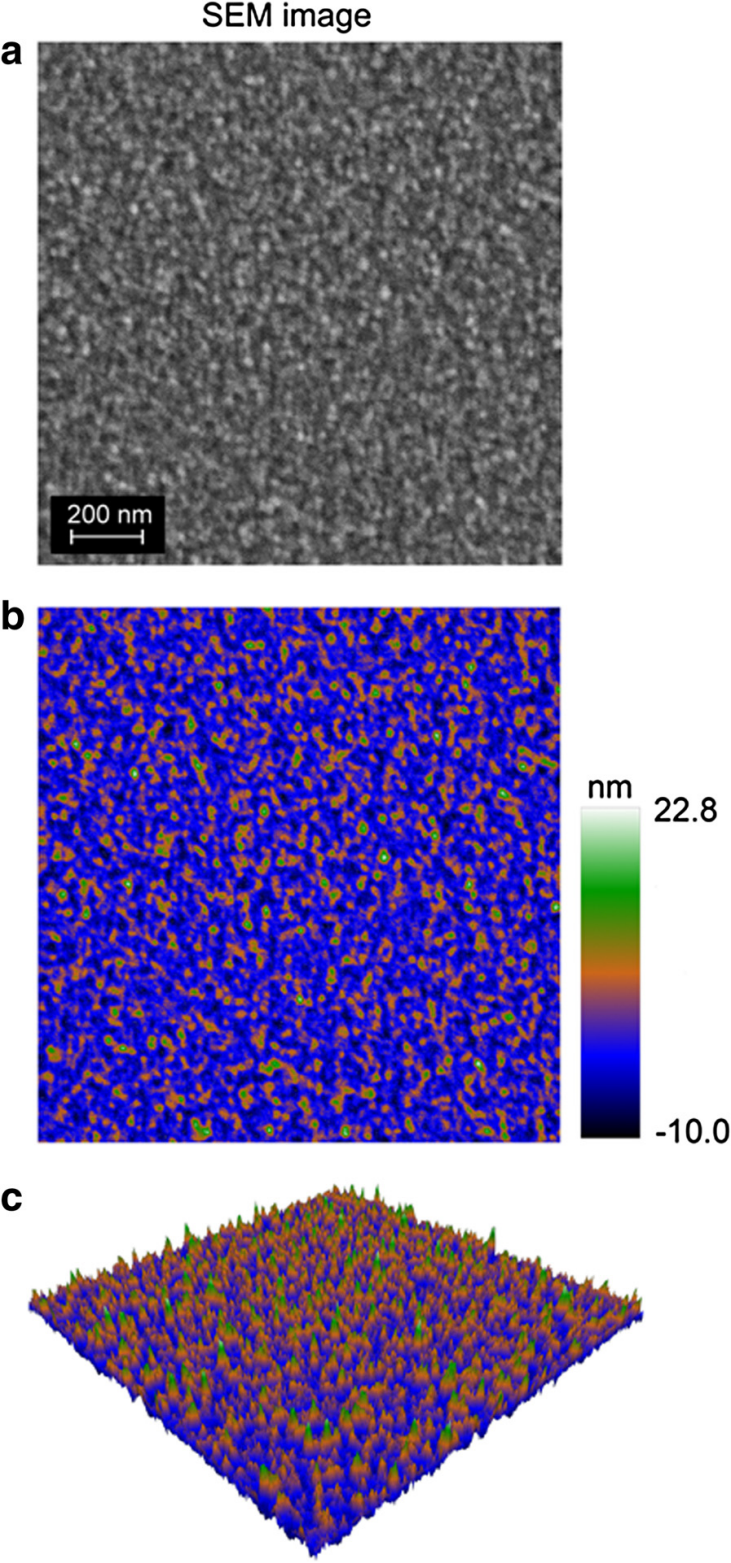
**Three-dimensional representation of a 150 nm-thick thin titanium film surface.** A section of an original scanning electron micrograph, 512 × 512 pixels in size was selected for conversion into a 3D object **(a)**. The image was assigned to a 2D polygonal plane as a displacement map, and a terrain color map was applied **(b)** before depth translation according to the pixel height values. Note that the color map presented in **(b)** has been calibrated through the use of AFM roughness data.

The process for generating the three-dimensional models from the base two-dimensional images was applied to scanning electron micrographs of titanium thin films, the data from which is presented in Figure 2. A planar co-ordinate mapping option, termed UV mapping, was applied on an axis that was perpendicular to the surface. UV mapping is the one of the most important parts of the 3D visualization process; its function is to create the two-dimensional texture coordinate system of the 3D model, which allows the placement of a 2D texture on a 3D model. A single co-ordinate was assigned to each vertex of the plane. The co-ordinates were applied after the resolution and dimensions were set.

A Lambert shading material was assigned to the polygonal plane. The type of material selected does not affect the displacement mapping. A new 2D file texture node was created and connected to the Lambert Material as a displacement map attribute. The file node allows the importation of an image into Maya. A filter type can be chosen for the image, which will affect the quality of the image. However, it will also have an effect on the 3D geometry shape when the image is converted into a 3D object. For the SEM image the Gaussian filter was selected. The filter will give the best image quality, resulting in a smooth 3D model. It did not affect the main features of the object.

There are 2 options in Maya for conversion of an image into a polygonal geometry. One is ‘convert - displacement to polygons’ and the other is ‘convert- displacement to polygons with history.’ The first option converts the image, however, it adds additional subdivisions to the surface and the resolution will be excessively high to process. This option is sufficient to convert polygonal objects with low starting subdivision value. The resulting 3D object was also very messy when the unnecessary resolution was added to it. The second option performs the same task, but only deforms the already defined subdivisions of the plane, without adding additional geometry. The conversion process also triangulates the subdivisions.

Three-dimensional animated movies were produced to aid in comparison between the models visualized via the displacement map technique (see Additional file 1). The 3D models of the same surface magnifications were presented with corresponding AFM scans (AFM left, SEM right), and rotated from 0° to 360° in tandem around the Y-axis in a time range of 15 seconds. The animation was rendered with the mental-ray plugin within Maya, and exported as sequential images in TARGA format. The rendered images were imported into Adobe Premier for post-production, where additional information was added, such as the average roughness values (*R*_a_) and the magnification values. The movie was then exported in MPEG file format.

## Results

### Visualization of titanium surfaces

**Figure 3:**
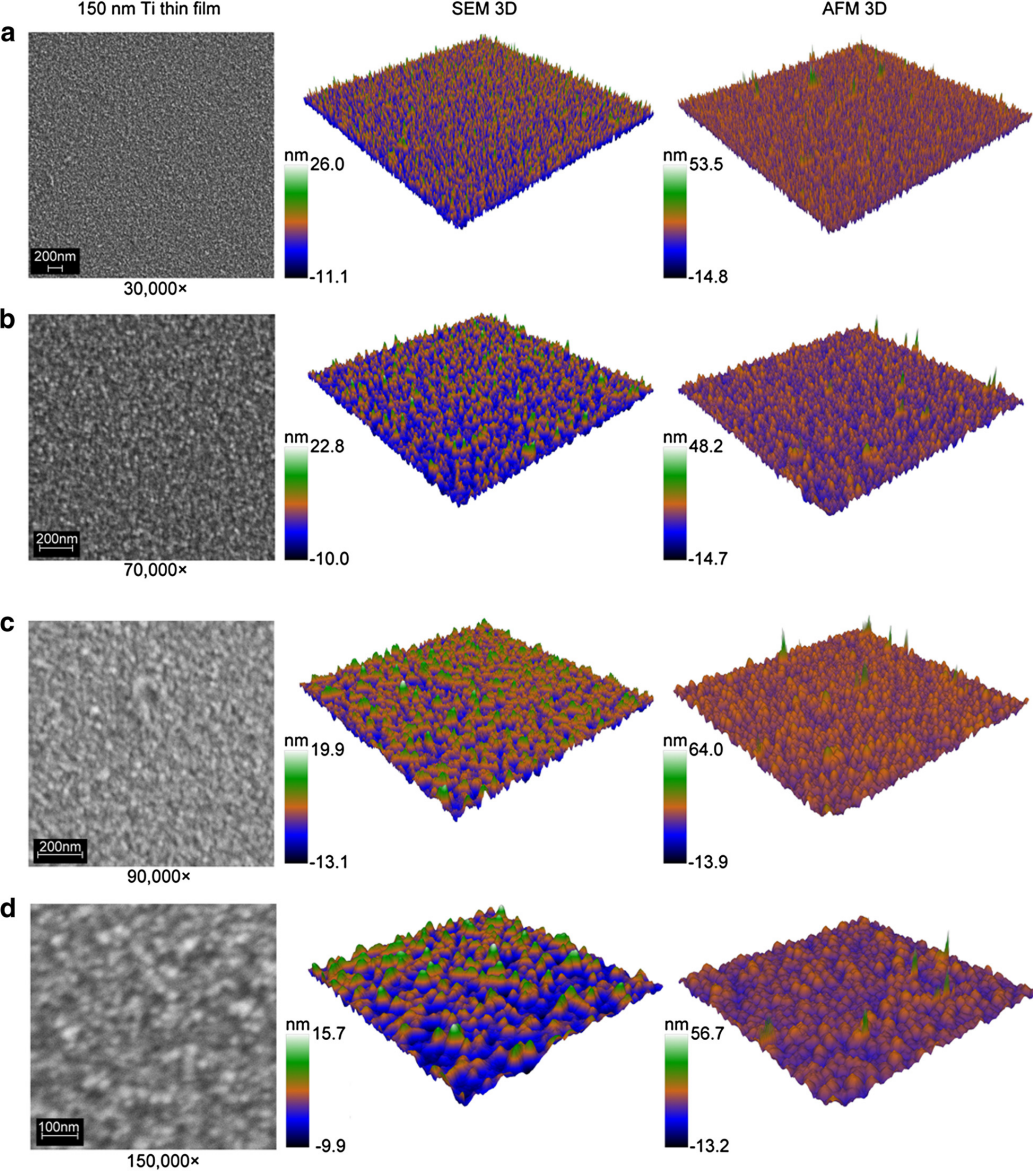
**Reconstruction of surfaces using input micrographs with various magnifications.** Three-dimensional surfaces have been generated based on electron micrographs of 150 nm-thick titanium thin films at 30000× **(a)**, 70000× **(b)**, 90000× **(c)** and 150000× **(d)** magnifications. Color scales have been calibrated via AFM roughness data. Three-dimensional surfaces generated based on AFM scans of corresponding surface areas are presented in the right column. The surface features in AFM scans appear to be more regular than in SEM 3D displacement maps, due to the effects of tip convolution on AFM data.

Three-dimensional displacement maps were successfully applied to scanning electron micrographs of the surface of 150 nm-thick sputter-coated titanium films, at 30000×, 70000×, 90000× and 150000× magnifications (Figure 3, Additional file 1). For each micrograph, a subsection was chosen that measured 512 × 512 pixels and was subsequently imaged via the displacement map technique. The height scale was calibrated using AFM scan data. Comparative surfaces generated from AFM scans are presented in the right-hand column of Figure 3. The surface features visible in the SEM displacement maps appear somewhat irregular, especially in comparison to those present in the 3D AFM surfaces. This difference can be attributed to one of the drawbacks associated with AFM as an imaging technique. The size and shape of the features on a sample imaged by AFM depend not only on the geometry of the features themselves but also that of the AFM tip that is used for the analysis. This effect is known as ‘tip convolution’, and while it can be mitigated to some degree through the use of sharper tips and deconvolution algorithms, it cannot easily be eliminated (Tabet and Urban, 1996; Shiramine et al., 2007). This is the advantage of SEM over AFM; by avoiding any physical interaction with the sample, a more accurate representation of the surface geometry can be obtained.

In the figures presented here, the height scale of the displacement maps was calibrated according to the AFM data. AFM analyses were performed on each of the samples visualized, and the average roughness (*R*_a_) was calculated. Average roughness is defined as the average deviation of the height values from the mean height (Stout et al., 1993; Brune et al., 1997; Webb et al., 2012). Each pixel within the displacement maps was assigned a proportional value between 0 and 1, therefore the average deviation from the mean of the pixel values was calculated, and the height values for each pixel were scaled so that the average deviation matched the *R*_a_ of the sample. It should be noted that the calculated *R*_a_ of a surface is dependent on the sampling interval, i.e. the resolution of the AFM scan, therefore scan areas were chosen in order to match the resolution of the AFM scans to that of the electron micrographs. Calibration of the height scales in this manner requires only a few time-consuming AFM scans, and the application of average roughness data can be applied to many SEM displacement maps. AFM is by no means the only technique available that can be used to calibrate the height scales; stylus and optical profilometers, for example, can also be used to record topographical data.

### Other materials and sample types

In addition to the titanium surfaces, three different substrata surfaces were used to produce displacement maps in order to assess the versatility of the technique. The three surfaces chosen were specifically selected for their highly diverse chemical compositions and surface topographies. The surfaces included dragonfly wings, unmodified silicon wafers, and polystyrene (PS) plastic excised from standard cell culture Petri plates (Figure 2). Dragonfly wings are known to have relatively large surface features (Ivanova et al., 2013; Nguyen et al., 2013), while polystyrene and silicon wafers are known to be quite smooth (Decuzzi and Ferrari, 2010; Gentile et al., 2010; Zeiger et al., 2013), and all three samples are less conductive than the previously utilized metallic titanium. In the case of dragonfly wings, the SEM displacement maps provide a clear advantage over AFM scans. The large feature size, and the inherent ‘stickiness’ of the epicuticular lipids that are present on the surface of the wing make it quite difficult to produce accurate AFM scans that are free from artifacts. The SEM displacement map presents a clearly defined structure. The SEM displacement map and AFM scan of the silicon surface are also relatively comparable; both techniques produce a smooth, feature-less surface.

**Figure 4:**
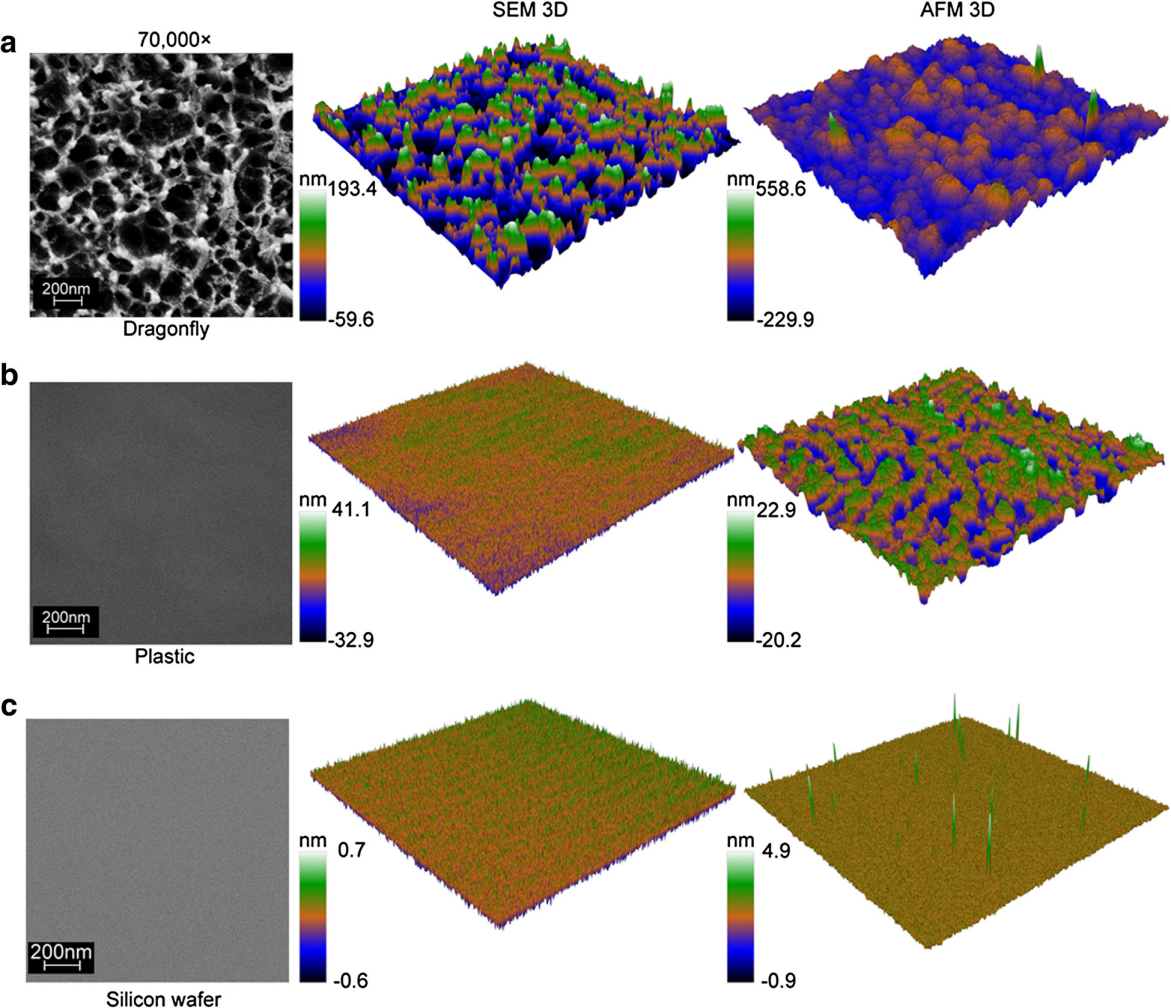
**Visualization of material surfaces with different compositions.** Three-dimensional surfaces of dragonfly (*Hemianax papuensis*) wings **(a)**, polystyrene Petri plates **(b)** and silicon wafers **(c)** can be reconstructed from electron micrographs. Magnification in all micrographs is 70000×, and color scales have been calibrated using AFM roughness data.

In contrast, little correlation can be drawn between the SEM displacement map and AFM scan of the PS surface. The well-defined topography on the PS surface (post gold coating) that was detected via AFM analysis was not represented well in the SEM displacement map (Figure 4b). This is largely due to the highly-insulating nature of the PS surface. Build-up of electrons on the surface of the sample decreases the resolution and depth of analysis, and homogenizes the resulting micrographs (Joy and Joy, 1996).

## Discussion

The advantage of this displacement map technique is in its ability to quickly generate 3D model representations of surfaces that can be more easily analyzed. In contrast to previous studies involving stereoimaging (Cuijpers et al., 2011), only a single SEM micrograph is required for the reconstruction of the 3D model. An additional advantage of using the displacement map technique is that complex mathematical computations are not required for the visualization process (Marschall et al., 2000; Samak et al., 2007). As a result, topographical surveys can be performed quickly, bearing in mind that the depth dimension of the data is an approximation.

To illustrate the advantages and limitation of the displacement map technique for generating 3D maps, consider the following example. An aerial-view photograph is recorded of a room full of people with a distribution of heights, colored in grey-scale according to the heights of each person. It is not a simple task to draw conclusions on the relative heights of different people, especially without reference to the color scale, however, it is of course much easier to obtain knowledge on the relative heights of the people when viewing from the side. The extra dimension allows the potential for insight into the height distribution to increase. The absolute heights of each individual could also be obtained by directly measuring them, however if this were time consuming the heights of a subsection of the people in the room could be measured, and provided that the distribution of heights throughout the population were homogeneous and random, data from that subsection could be applied to approximate the heights of the rest of the group. Thus, the relative heights of the people are accurately represented, and their absolute heights are approximated in a short time. Provided one keeps in mind the limitations of the technique, this can be a highly effective tool in analyzing large populations of data.

**Figure 5:**
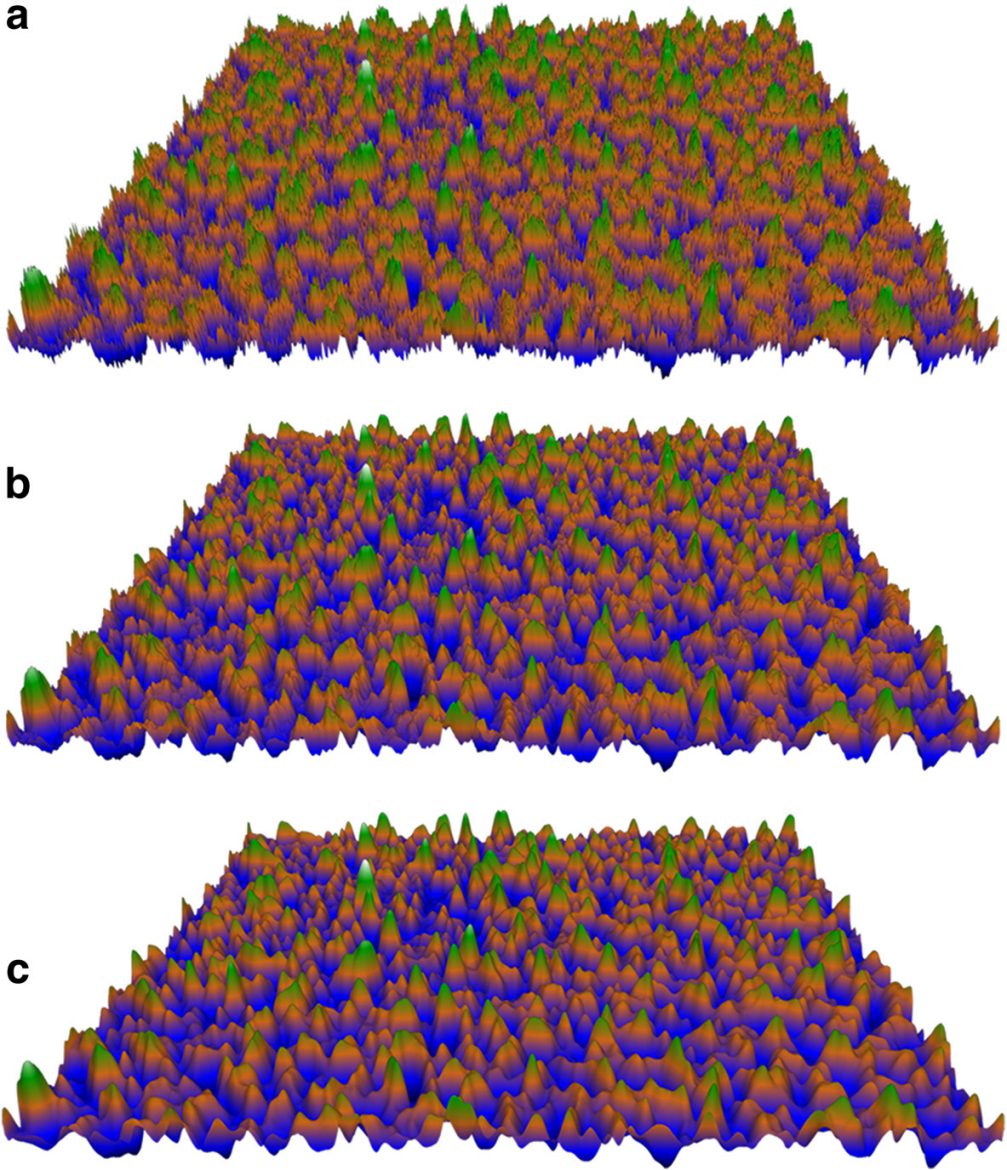
**SEM noise reduction for surface reconstruction.** A high level of noise in an electron micrograph can adversely affect the final 3D geometry, resulting in a highly rough appearance **(a)**. In order to minimize the effect of noise, a convolution algorithm can be applied. The surface presented in **(b)** was processed using a 3 × 3 convolution matrix to ‘smooth out’ the effect of noise. Care must be taken however, to ensure that the image is not excessively smoothed, as this will result in the loss of topographical detail. This is demonstrated in surface **(c)**, where the same convolution matrix was applied 5 times in succession.

There are some limitations associated with the use of displacement maps to visualize SEM images in three dimensions. Firstly, given that the displacement maps are generated from two-dimensional data, only the uppermost features can be visualized. For example, if one feature lies below another, e.g. if the angle of one larger feature causes it to overshadow a nearby smaller feature, then the lower feature cannot be visualized. As much as possible, the scan data should be collected from an angle of 90°, perpendicular to the plane of the surface. Similarly, the quality of the three-dimensional displacement map will be dependent on the quality of the input micrograph (Cizmar et al., 2008). Typically, SEM software allows for the manipulation of the brightness and contrast prior to the capture of the image. Ideally, for the application of displacement maps, the contrast should be set to the maximum level possible without losing the topological detail in the upper and lower extremities of the surface. Also as with any experimental technique, there is a requirement to minimize the noise with respect to the collected signal (Hiraiwa and Nishida, 2012b; 2012a). For example, an electron micrograph with high noise levels will typically contain many bright ‘dots’, which will subsequently appear as very tall and very narrow features in the corresponding displacement map. An example of a displacement map created from a noisy micrograph is presented in Figure 5a. Fortunately, this effect can often be remedied post-data collection via the application of smoothing filters. A smoothing filter adjusts the pixel values to the average of the surrounding pixels, after weighting each pixel according to a user-defined matrix. The size of the matrix, the weighting of the pixels, and the number of times the filter is applied is completely customizable to enable the production of the best image possible (Figure 5b). One must be careful however, not to excessively smooth the image, as this will likely cause the topographical details to be lost (Figure 5c).

Finally, the results presented here demonstrate that displacement maps cannot always be applied to the surfaces of insulating materials. Samples that are weakly or moderately insulating can be coated with a thin layer of gold or carbon, which is common practice in electron microscopy (Wang et al., 2008). This process improves the conductivity of the surface and enables micrographs of adequate quality to be obtained. The thickness of the conductive coating must be minimized as much as practically possible in order to avoid losing surface details (Nowell and Pawley, 1980). For strongly insulating materials however, electron micrographs do not contain sufficient topographical detail to produce useful 3D displacement maps.

The ability to produce three-dimensional displacement maps based on two-dimensional data widens the range of analytical techniques that can be applied for the visualization and assessment of surface topography. This technique is particularly applicable to scanning electron micrographs, when calibrated appropriately using data collected from AFM scans. Displacement maps are particularly effective for visualizing conductive surfaces such as titanium, or for viewing surfaces that cannot be easily examined by other topography analysis tools, such as AFM.

## Competing interest

The authors declare that they have no competing interests.

## Authors’ contribution

VB carried out the 3D visualization of SEM images, HKW and VTHP conducted AFM scans and calibration, VTHP carried out SEM, EPI, CJF designed experiments, all authors participated in data analysis; VB, HKW, EPI and RJC drafted the manuscript. All authors read and approved the final manuscript.

## Acknowledgements

This study was supported in part by Australian Research Council (ARC). Autodesk, Maya are registered trademarks or trademarks of Autodesk, Inc., and/or its subsidiaries and/or affiliates in the USA and/or other countries.

## Supplementary Material

Additional file 1 **Three-dimensional surface models of titanium thin films, dragonfly wings and silicon wafer based on two-dimensional data.** Comparative visualization of material surfaces of 150 nm-thick titanium thin films, dragonfly (*Hemianax papuensis*) wings and silicon wafers derived from atomic force microscopy (AFM) roughness data and scanning electron microscopy (SEM) imaging. Click here for file
